# Substrate-mediated effects in photothermal patterning of alkanethiol self-assembled monolayers with microfocused continuous-wave lasers

**DOI:** 10.3762/bjnano.3.8

**Published:** 2012-01-26

**Authors:** Anja Schröter, Mark Kalus, Nils Hartmann

**Affiliations:** 1 Fakultät für Chemie, Universität Duisburg-Essen, 45117 Essen, Germany; 2 Center for Nanointegration Duisburg-Essen (CENIDE), Universität Duisburg-Essen, 47048 Duisburg, Germany

**Keywords:** femtosecond lasers, nonlinear laser processing, self-assembled monolayers, subwavelength patterning, ultrathin resists

## Abstract

In recent years, self-assembled monolayers (SAMs) have been demonstrated to provide promising new approaches to nonlinear laser processing. Most notably, because of their ultrathin nature, indirect excitation mechanisms can be exploited in order to fabricate subwavelength structures. In photothermal processing, for example, microfocused lasers are used to locally heat the substrate surface and initiate desorption or decomposition of the coating. Because of the strongly temperature-dependent desorption kinetics, the overall process is highly nonlinear in the applied laser power. For this reason, subwavelength patterning is feasible employing ordinary continuous-wave lasers. The lateral resolution, generally, depends on both the type of the organic monolayer and the nature of the substrate. In previous studies we reported on photothermal patterning of distinct types of SAMs on Si supports. In this contribution, a systematic study on the impact of the substrate is presented. Alkanethiol SAMs on Au-coated glass and silicon substrates were patterned by using a microfocused laser beam at a wavelength of 532 nm. Temperature calculations and thermokinetic simulations were carried out in order to clarify the processes that determine the performance of the patterning technique. Because of the strongly temperature-dependent thermal conductivity of Si, surface-temperature profiles on Au/Si substrates are very narrow ensuring a particularly high lateral resolution. At a 1/e spot diameter of 2 µm, fabrication of subwavelength structures with diameters of 300–400 nm is feasible. Rapid heat dissipation, though, requires high laser powers. In contrast, patterning of SAMs on Au/glass substrates is strongly affected by the largely distinct heat conduction within the Au film and in the glass support. This results in broad surface temperature profiles. Hence, minimum structure sizes are larger when compared with respective values on Au/Si substrates. The required laser powers, though, are more than one order of magnitude lower. Also, the laser power needed for patterning decreases with decreasing Au layer thickness. These results demonstrate the impact of the substrate on the overall patterning process and provide new perspectives in photothermal laser patterning of ultrathin organic coatings.

## Introduction

In the past decades, self-assembled monolayers (SAMs) have developed into a particularly versatile means to tailor the surface properties of technologically important materials, such as gold, silicon and glass [1–3]. Because of the self-limiting growth mechanism, well-defined coating with a layer of monomolecular thickness is ensured [4]. Varying the chemical structure of the precursor molecules, in turn, allows one to alter the chemical reactivity and resistance of these coatings [5]. These characteristics of SAMs have been widely exploited in numerous micro- and nanofabrication schemes [1–3]. A prominent example, addressed here, considers the application of SAMs as ultrathin resists. Patterning techniques, such as scanning-probe techniques, e-beam lithography, micro-contact printing and photolithography have been employed along this path [6–9]. Furthermore, laser processing of SAMs has attracted significant attention [9–12]. Generally, laser techniques provide a variety of powerful features and hence are the preferred choice in many technical and medical applications [13]. Prominent examples include optical data storage, photo-mask fabrication and manufacturing of medical implants [14]. Owing to the optical diffraction limit, laser nanofabrication encounters significant challenges. Typically, minimum structure sizes are not much smaller than the wavelength of the laser source [13]. A means to extend the lateral resolution of laser patterning techniques into the subwavelength range is to take advantage of nonlinear effects, such as photothermal and multiphoton absorption processes [11–17]. In photothermal processing, laser light is used in order to locally heat the substrate surface and initiate chemical reactions [12]. Commonly, photothermal patterning of SAMs is carried out by sequential processing with microfocused lasers [11,18–25]. In addition, some contributions also demonstrated parallel processing through the use of microlens arrays and interference patterns [26–27]. These contributions emphasize the prospects of photothermal laser routines in micro- and nanopatterning of different types of SAMs and other ultrathin organic coatings [11,18–28]. Because of the photothermal process, the performance of such laser techniques depends on both the peculiar chemical structure of the SAM, notably the surface linkage, and the optical and thermal properties of the substrate [11,13]. In this contribution we focus on substrate-mediated effects in photothermal laser patterning of alkanethiol SAMs on Au-coated Si and glass substrates. Patterning experiments are combined with temperature calculations and thermokinetic simulations. Although photothermal patterning of alkanethiol SAMs on distinct substrates has been investigated previously [11,21–24,27], a systematic study on the influence of the substrate on the performance of the patterning technique is still missing. The results reported here demonstrate a strong dependence of the patterning process on the support material, i.e., on its thermal conductivity. Comparative experiments with Au-coated glass substrates also show a strong impact of the Au layer thickness.

## Results and Discussion

### General approach

**Figure 1:**
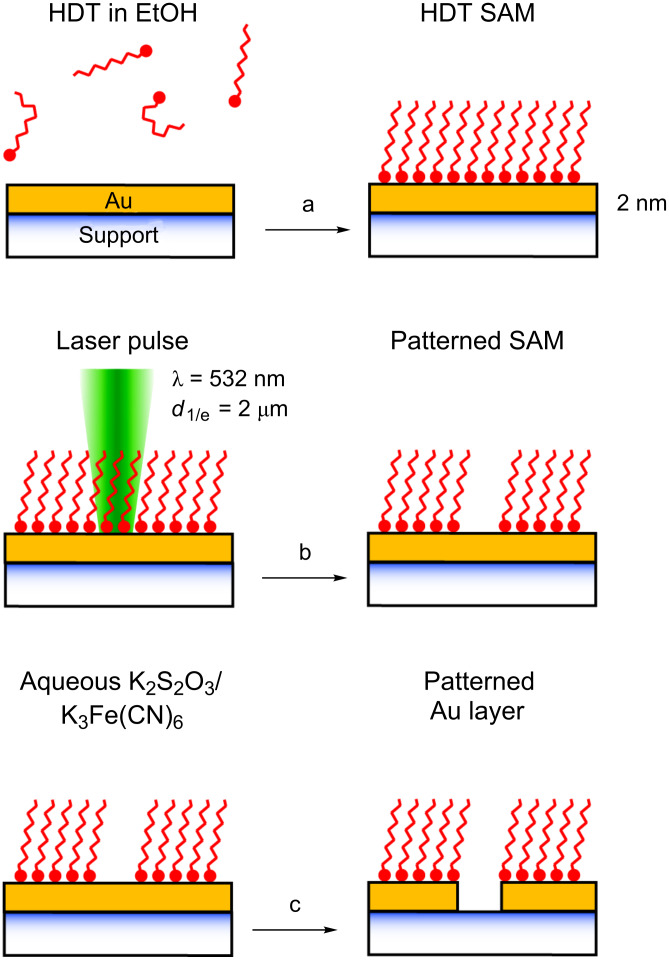
Schematic diagram of the process flow: (a) SAM formation upon immersion in an ethanolic solution of HDT; (b) photothermal laser processing of the HDT SAM at λ = 532 nm; and (c) pattern transfer into the Au film upon etching in an aqueous solution of K_2_S_2_O_3_ and K_3_Fe(CN)_6_. Adapted from [11].

The general experimental approach is illustrated in Figure 1. Alkanethiol SAMs were prepared by immersion of Au-coated glass and silicon substrates into a millimolar solution of hexadecanethiol (HDT). Photothermal processing was carried out by using a microfocused laser beam at λ = 532 nm and *d*_1/e_ = 2 µm. The experimental setup allows the variation of the laser power *P* and the laser pulse length τ. In a patterning experiment the sample was moved in the focal plane of the laser. This provides a convenient means to test distinct laser parameters in adjacent surface areas. At sufficiently high laser powers and/or sufficiently long pulse lengths, thermal desorption of the thiol molecules is initiated [11]. Subsequently, the Au layer in these laser-depleted surface areas was removed by means of wet-chemical etching [11,29]. For this purpose, the patterned substrates were immersed into an aqueous solution of K_2_S_2_O_3_ and K_3_Fe(CN)_6_. The HDT SAM acts as an ultrathin resist and inhibits etching in the coated surface areas. The immersion time was adjusted in order to completely dissolve the Au film in the laser-depleted surface areas and to minimize widening of the structures owing to the isotropic etching process.

### Characterization of substrates and monolayers

**Figure 2:**
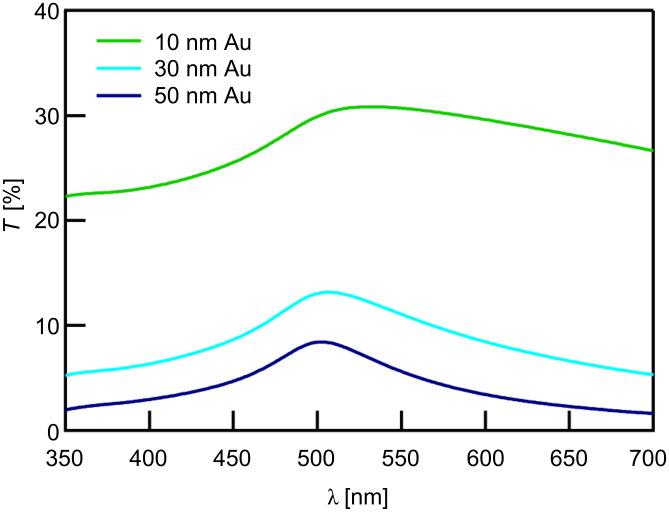
UV–vis spectra of Au/glass substrates with Au layer thicknesses of 10 nm, 30 nm and 50 nm.

As substrates, Au-coated glass plates with Au layer thicknesses of 10 nm, 30 nm, 50 nm and 100 nm were used. In addition, experiments with Au-coated silicon substrates with a 30 nm Au layer were carried out. UV–vis spectra of Au-coated glass supports are displayed in Figure 2 [30–31]. Evaporated Au films with a thickness of 10 nm or below often exhibit a discontinuous structure and show a plasmon resonance in the UV–vis spectrum, that is, a pronounced minimum in the spectral transmission between 500 and 600 nm. This plasmon resonance is not observed here, suggesting that all substrates exhibit a continuous Au layer. Atomic force microscopy (AFM) revealed a surface roughness of a few nanometers. Note that Au/glass substrates with 100 nm thick Au layers and Au/Si substrates are opaque and, hence, do not allow for characterization by means of UV–vis spectroscopy. In addition, the transmittance *T* and reflectance *R* at a wavelength of 532 nm and normal incidence was determined. The respective data are summarized in Table 1. Taking into account the transmittance and reflectance data allows one to calculate the absorbance *A* and the effective absorption coefficient α_Au_ of the films from [12]:

[1]
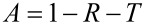


and

[2]
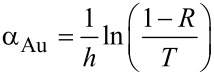


It is worth noting that substrates with thin Au layers exhibit the highest absorbance; the optical data for glass substrates with 100 nm thick Au layers, in turn, correspond to the bulk values of Au [32]. For comparison, the 1/e penetration depth of bulk Au at a wavelength of 532 nm is about 18 nm only [32].

**Table 1:** Optical properties of Au-coated substrates at λ = 532 nm.

Support	*h*_Au_ [nm]	*R*	*T*	*A*	α_Au_ [cm^–1^]

Glass	10	0.34	0.31	0.35	7.56·10^5^
Glass	30	0.61	0.12	0.27	3.90·10^5^
Glass	50	0.68	0.07	0.25	3.08·10^5^
Glass	100	0.75	0.00	0.25	5.69·10^6,a^
Si	30	0.71	0.00	0.29	—^b^

^a^Given value refers to the bulk value for Au [32]. ^b^Indeterminable because of the opacity of the Si support.

**Figure 3:**
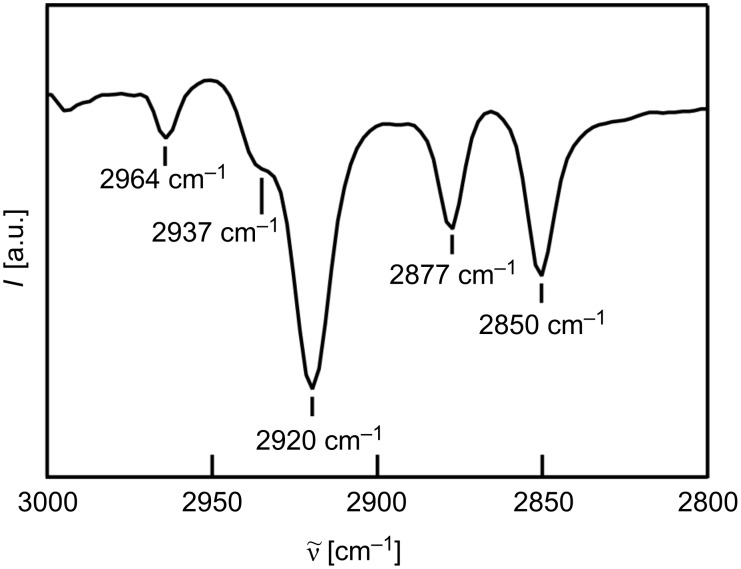
IRRAS-spectra of an HDT-coated Au/glass substrate exposing a 50 nm Au layer.

HDT coated substrates are characterized by contact-angle measurements and infrared reflection–absorption spectroscopy (IRRAS). Static water contact angles are about 109°. IR measurements show no difference for all samples considered here. A typical spectrum is shown in Figure 3, and peak assignments are given in Table 2. Based on the peak positions of the antisymmetric methylene stretching vibrations, these data indicate densely packed monolayers [33].

**Table 2:** Assignment of IR peaks.^a^

Peak	Position [cm^–1^]

ν_as_(CH_3_)_ip_	2964
ν_s_(CH_3_)_FR_	2937
ν_as_(CH_2_)	2920
ν_s_(CH_3_)_FR_	2877
ν_s_(CH_2_)	2850

^a^ν_s_ and ν_as_ refer to the symmetric and antisymmetric stretching vibrations; ip refers to in-plane vibrations; FR indicates vibrations which are split because of Fermi resonance interactions with lower-frequency vibrations [33].

### Photothermal laser patterning

**Figure 4:**
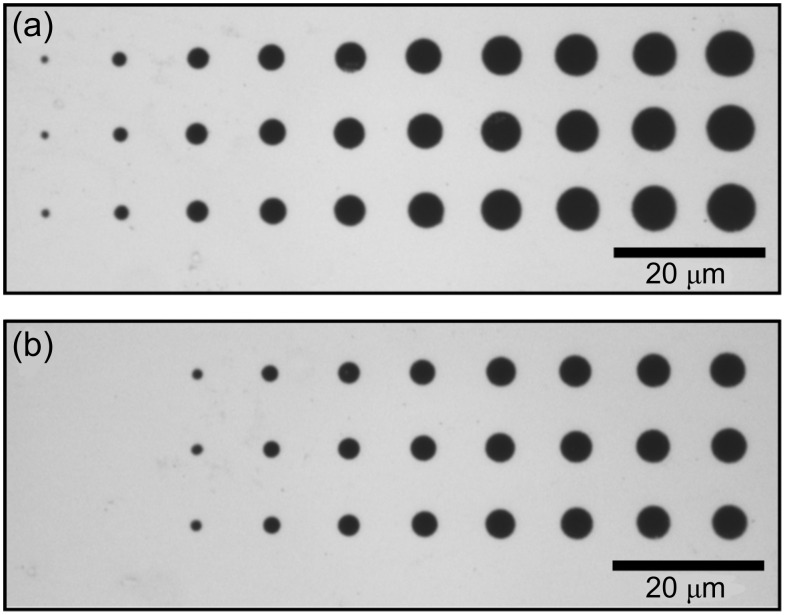
Optical micrograph of a laser-fabricated dot pattern. HDT-SAMs on a Au/glass substrate exposing a 30 nm thick Au layer are processed with single laser pulses with distinct τ between 50 µs (left) and 10 ms (right) and (a) *P* = 24.3 mW, (b) *P* = 20.3 mW. After laser processing, the pattern is transferred to the Au layer by wet-chemical etching.

**Figure 5:**
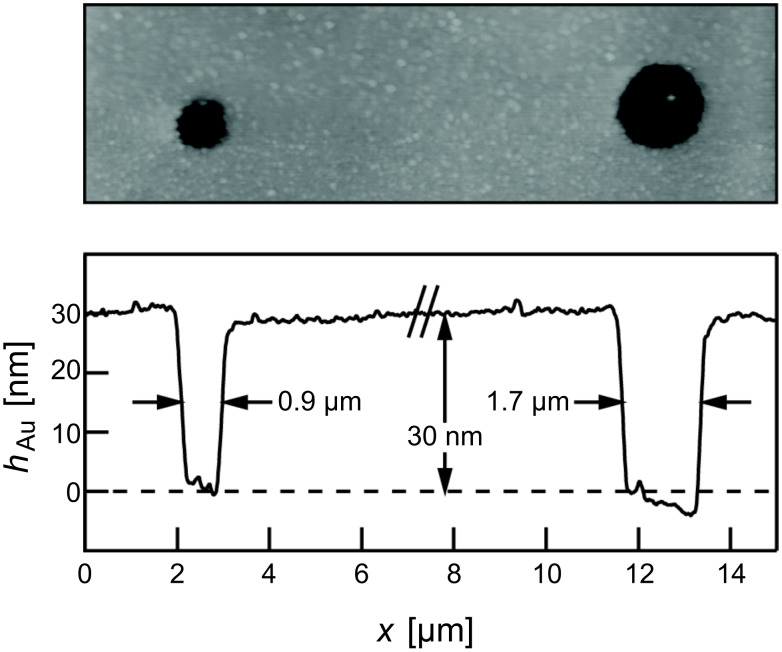
AFM data from patterning experiments with HDT-SAMs on Au/glass substrates exposing a 30 nm thick Au layer. The structures were fabricated by using single laser pulses at *P* = 24.3 mW and with distinct τ of 50 µs (left) and 100 µs (right). Pattern transfer to the Au layer was carried out by wet-chemical etching. Diameters refer to values at half-depth.

After the etching process, patterned samples were characterized by optical microscopy. Typical micrographs of patterns on a glass support are shown in Figure 4. Each micrograph displays a pattern that has been fabricated at a given laser power and with distinct laser pulse lengths between 50 µs and 10 ms. In order to check the reproducibility, the patterning was carried out under identical conditions along three rows. For precise characterization of the structures, atomic force microscopy (AFM) was used. Figure 5 displays a topographic AFM image and a height profile of structures with diameters of 0.9 µm and 1.7 µm. The depth of these structures is equivalent to the thickness of the Au layer of 30 nm. Diameters are measured at the half depth. Because of the isotropic etching process, these values are expected to be slightly larger than the diameter of the depleted areas after laser processing. Considering a 30 nm thick film, for example, the widening at the half depth amounts to about ±15 nm. For all structure sizes reported here this is <<10% of the total width. Hence, this effect is considered to be negligible and is not taken into account. Note also, that the measurements are not corrected for the tip size. Hence, the measured diameters, indeed, are somewhat smaller than the actual width of the structures. This to some extent compensates for the widening of the structures during etching.

**Figure 6:**
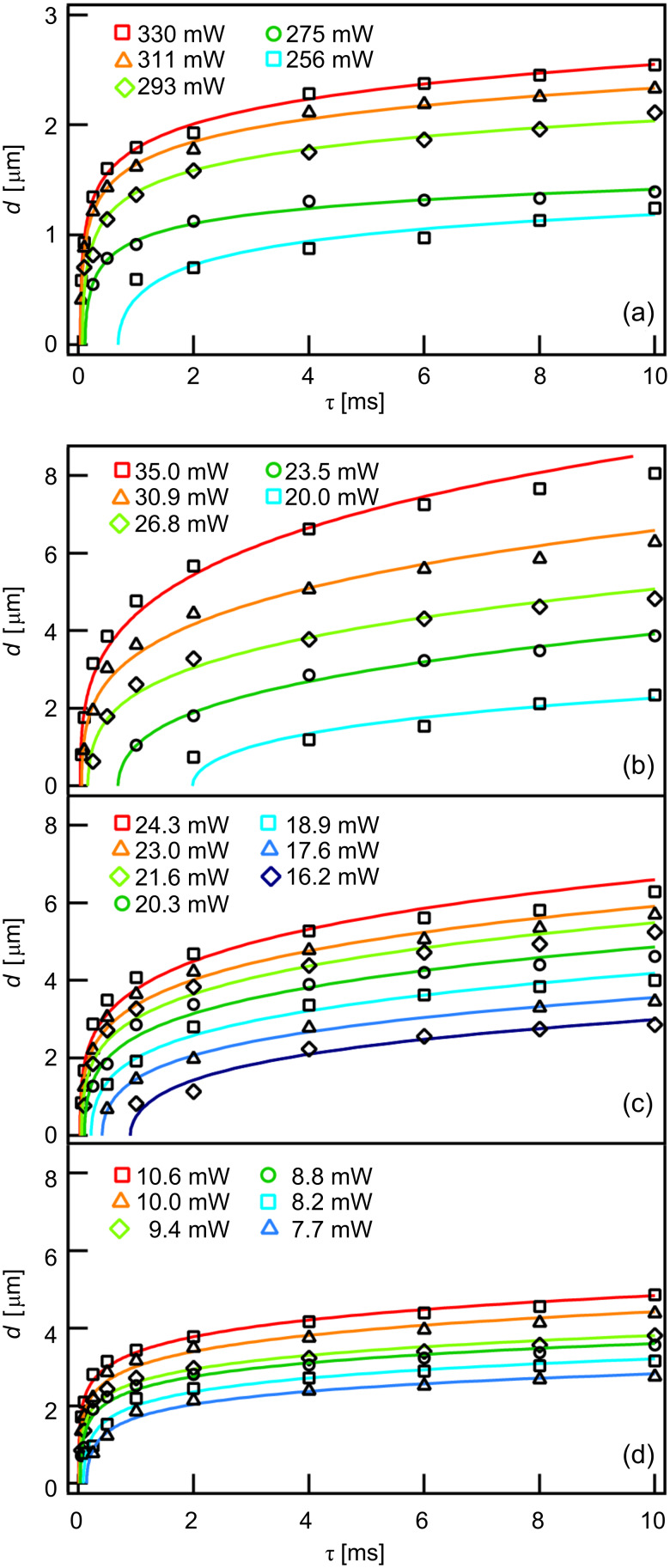
Dependence of the structure diameter *d* on the incident laser power *P* and the pulse length τ of HDT-SAMs on (a) Au/Si substrates with a 30 nm Au layer, (b) Au/glass substrates with a 50 nm Au layer, (c) Au/glass substrates with a 30 nm Au layer and (d) Au/glass substrates with a 10 nm Au layer. The lines are guides for the eyes only.

Figure 6 displays the dependence of the structure diameter *d* on the laser parameters. In order to ensure comparability, only data from structures exhibiting a depth that is equivalent to the respective Au layer thickness are considered. Complete etching of the laser-depleted areas on patterned substrates with a 100 nm thick Au layer turned out to be difficult. Hence, no data for such samples are shown. All diagrams display the typical dependence of the structure diameter on the laser power and laser pulse length, as observed in a previous study focusing on photothermal patterning of HDT-SAMs on Au/Si substrates [11]. It is noteworthy, though, that processing of HDT-SAMs on Au/glass substrates can be carried out at much lower laser powers. Photothermal patterning of alkanethiols on Au/glass substrates at low laser powers has been reported previously [21,24]. Due to the different experimental parameters, however, a quantitative comparison of these data is not feasible. The data presented here demonstrate that, under otherwise identical conditions, the laser powers needed for patterning of HDT-SAMs on glass supports are reduced by more than one order of magnitude when compared with those values needed for patterning of HDT-SAMs on Si supports. Moreover, when comparing the data on the Au-coated glass substrates, a strong dependence of the patterning results on the Au layer thickness is evident. The average laser power required for fabrication of identical structures decreases from 28 mW to 9 mW when the Au layer thickness is reduced from 50 nm to 10 nm. Patterning of HDT-SAMs on Au/glass supports with Au layer thicknesses of 10 nm can be carried out at laser powers below 8 mW, a value comparable with the emitted power of a laser pointer. This opens up an opportunity for truly cost-effective laser processing of thiol-based SAMs. In addition, parallel processing, e.g., by using micromirror displays [34], appears feasible.

The choice of the support material, of course, also affects the lateral resolution of the laser technique. Processing of HDT-SAMs on Au/Si substrates can be carried out with a high lateral resolution. In particular, structure sizes are much smaller when compared with the data from equivalent patterning experiments with Au/glass substrates (Figure 6). Minimum structure sizes on Si supports are between 300 and 400 nm. This is somewhat larger when compared with those values in the range of 200–300 nm that were obtained with a very similar laser setup [11]. Structure sizes on Au/glass substrates, in turn, decrease with decreasing Au layer thickness. However, irrespective of the Au layer thickness, the smallest structures on glass supports exhibit a width between 600 and 700 nm, which is to say that no correlation between the achievable minimum structure size and the Au layer thickness is evident. For comparison, in a previous study focusing on photothermal patterning of alkanethiol SAMs on Au/glass substrates, by using high-aperture immersion optics, minimum structure sizes in the range of 400–500 nm were reported [24].

### Temperature calculations

All patterning experiments described here were carried out with HDT-coated substrates. Hence, the distinct experimental observations are attributed to the peculiar optical and thermal properties of the Au-coated supports. This, of course, affects the temperature rise on the substrate surface and, hence, is well expected to influence the overall patterning process. Commonly, in photothermal processing with microfocused lasers the local temperature rise is calculated by considering the underlying heat-conduction equation [12]. Constant surface temperatures are rapidly established. Hence, for pulse lengths in the micro- or millisecond range, stationary temperature profiles *T*(*r*) are considered, where *r* corresponds to the radial position relative to the center of the laser spot [11]. The following paragraphs detail how the surface temperature profiles are calculated for Au/Si and Au/glass substrates. A description of all parameters and constants, as introduced in the following, is given in Table 3.

**Table 3:** Parameters and constants used in temperature calculations and thermokinetic simulations.

Description	Symbol	Value

Laser spot diameter at 1/e	*d*_1/e_	2 µm
Incident laser power	*P*	see Figure 7
Laser pulse length	τ	see Figure 7
Sample reflectance	*R*	see Table 1
Thermal conductivity of Au^a^	κ_Au_	3.15 W·cm^–1^·K^–1^
Thermal conductivity of glass^a^	κ_glass_	1.2·10^–2^ W·cm^–1^·K^–1^
Thermal conductivity of Si at *T*_0_^a^	κ_Si_	1.48 W·cm^–1^·K^–1^
Basic sample temperature	*T*_0_	300 K
Fit parameter for Si^a^	*T*_k_	96 K
Absorption coefficient of Au^b^	α_Au_	see Table 1
Au layer thickness	*h*_Au_	see Table 1
Activation energy^b,c^	*E*_A_	145 kJ·mol^–1^
Frequency factor^b,c^	ν	1.1·10^18^ s^–1^
Ideal gas constant	*R*_G_	8.314 J·K^–1^·mol^–1^

^a^[12]. We note that the thermal conductivity of thin Au films is generally lower when compared with the bulk value for Au. The exact value depends on the film thickness and on the specific film structure, which, in turn, varies depending on the detailed preparation procedure. Hence, widely varying thermal conductivities are discussed in the literature [35]. For simplicity, the bulk value is considered here. Very similar results are obtained with lower thermal conductivities. ^b^Given parameters refer to effective parameters. ^c^[11].

In the case of Au/Si substrates, laser absorption largely takes place in the thin Au layer, whereas heat conduction is dominated by the underlying Si support. This allows the calculation of the respective surface-temperature profiles on the basis of an analytical solution of the underlying heat-conduction equation considering surface absorption [11–12]:

[3]
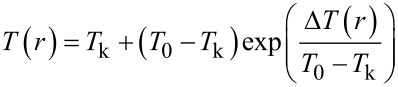


with

[4]
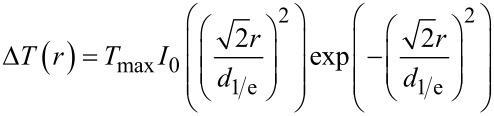


and

[5]
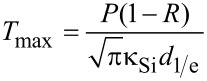


Note, *I*_0_, here and in the following, denotes the modified Bessel function of order zero. Moreover, Equation 3 takes into account the temperature-dependent thermal conductivity of Si.

In the case of Au/glass substrates laser absorption is strictly limited to the thin Au layer. Hence, again surface absorption applies. In contrast to Si, however, glass exhibits a very low thermal conductivity. For this reason, heat conduction is strongly affected by the Au layer. An approach reported by Calder and Sue allows one to take this into account and numerically calculate respective surface temperature profiles [36]. Considering a Gaussian beam and the dimensionless parameters r* = 2*r*/*d*_1/e_, κ* = κ_Au_/κ_glass_, α_Au_* = α_Au_*d*_1/e_/2, and h* = 2*h*_Au_/*d*_1/e_, the surface temperature profiles *T*(*r*) are given by [12,35]:

[6]
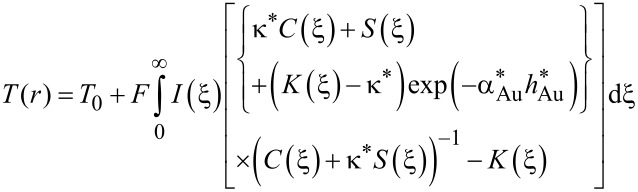


with

[7]
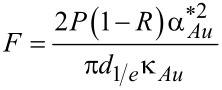


[8]
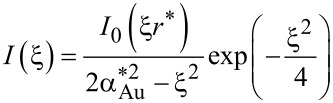


[9]
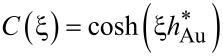


[10]
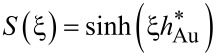


and

[11]
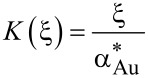


Despite certain approximations, calculations on the basis of Equations 3–5 and Equations 6–11 provide reasonable estimates of the surface-temperature profiles on the distinct substrates considered here [11–12,36]. This offers insights into the processes that determine the performance of the patterning technique.

**Figure 7:**
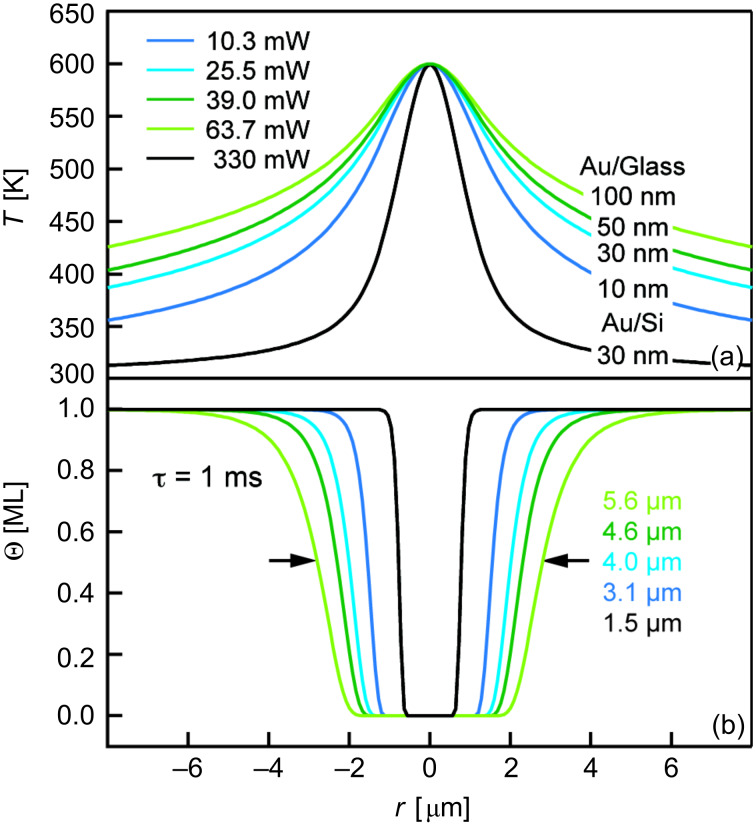
(a) Calculated stationary temperature profiles on different types of Au-coated substrates used for the patterning experiments. Laser powers *P* were adjusted in order to reach the same peak temperature of 600 K. Values are given in the diagram. (b) Corresponding surface coverage profiles at a laser pulse length of τ = 1 ms.

To illustrate the impact of the distinct substrate structure on the local temperature rise, surface-temperature profiles exhibiting the same peak temperature of 600 K are shown in Figure 7. Two general effects are evident from these data. Firstly, the laser power required in order to establish a certain peak temperature on Au/glass substrates is much lower than that needed for Au/Si substrates. Moreover, on glass supports, the required laser power strongly decreases with decreasing Au layer thickness. Secondly, the width of the temperature profile is much broader on glass supports and increases with increasing Au layer thickness.

Generally, in photothermal laser processing the peak temperature *T*_max_ is proportional to the absorbed laser power *P*_Abs_ and inversely proportional to the thermal conductivity of the substrate κ, that is *T*_max_ 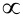 *P*_Abs_/κ [12]. As evident from Table 1, the absorbances of the substrates all are of comparable magnitude. The thermal conductivities of the supports, in turn, strongly vary (Table 3). In particular, depending on the specific temperature, the thermal conductivity of Si is one to two orders of magnitude larger than the thermal conductivity of glass [12]. Hence, the strong difference in the laser power required in order to reach a certain peak temperature rise is attributed to the largely distinct heat dissipation in the supports, Si versus glass.

Au, of course, exhibits a very high thermal conductivity. Thus, with increasing Au layer thickness thermal conduction in Au/glass substrates is more and more affected by heat dissipation within the Au film and the laser power required in order to establish a certain peak temperature increases.

The distinct thermal properties of the substrates also determine the width of the temperature profiles. The width of the temperature profiles on Au/Si substrates is determined by the Si support. Because of the temperature-dependent thermal conductivity of Si this results in a particularly narrow surface-temperature profile. Au/glass substrates, in turn, exhibit a strong difference in lateral and vertical heat conduction. Lateral heat conduction within the Au film is much faster than vertical heat conduction into the bulk of the support. For this reason, surface temperature profiles on Au/glass substrates are much broader when compared to those on Au/Si substrates. Also, with increasing Au layer thickness, in the range of 10 to 100 nm, lateral heat conduction increases. Hence, the width of the temperature profiles broadens.

### Thermokinetic simulations

Thermokinetic simulations are helpful to illustrate the impact of the surface temperature profiles *T*(*r*) on the diameter of the laser-fabricated structures. For this purpose, surface-coverage profiles θ(*r*) are calculated assuming first-order kinetics. A description of all parameters and constants, as introduced in the following, is given in Table 3. Due to rapid heating and cooling rates, the reaction time in photothermal laser processing essentially corresponds to the laser pulse length τ. Further details are discussed in a previous study [11]. Following this approach, surface coverage profiles θ(*r*) are calculated from

[12]
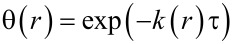


with *k*(*r*) denoting the radially varying reaction rate constant:

[13]
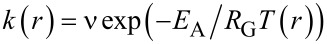


Considering Equation 12 and Equation 13, the local reaction kinetics depends on the irradiation time τ and the rate constant *k*(*r*)*,* which itself depends on the temperature. At a constant irradiation time, a certain temperature is required in order to induce substantial desorption of thiol molecules [21–22,37]. Following Equations 3–11 this necessitates a critical laser power density. Processing at short irradiation times demands high power densities, which may lead to complications, such as surface melting and substrate ablation. Hence, the procedure has to be carefully optimized in order to ensure selective processing of the SAM [11,24].

Calculated surface-coverage profiles at a typical laser pulse length of τ = 1 ms are displayed in Figure 7. Clearly, an increase in the diameter of the laser-depleted surface areas can be seen when comparing Au/Si to Au/glass substrates exposing Au layers of the same thickness. Also, for Au/glass substrates the diameters of the structures increase with increasing thickness of the Au layer. This is in agreement with the experimental data shown in Figure 6. Note that the structure diameter at short laser pulse lengths is ultimately determined by the width at the very top of the temperature profiles [11]. As evident from Figure 7a, this width is of comparable size for all Au/glass substrates considered here. For this reason, minimum structures on Au/glass supports are of comparable size irrespective of the Au layer thickness.

## Conclusion

Photothermal laser processing has developed into a valuable technique for the fabrication of micro- and nanostructured SAMs. The results presented here emphasize the impact of the substrate on the performance of this technique. In particular, the results of photothermal processing of thiol-based SAMs on Au/Si and Au/glass substrates, with Au layer thicknesses in the range of 10–50 nm, are compared. Minimum structure sizes are significantly smaller on Au/Si substrates. It is, however, worth noting that the processing of Au/glass substrates can be carried out at very low laser powers. In addition, the required laser power for patterning on Au/glass substrates strongly decreases with decreasing Au layer thickness. This opens up new perspectives in low-cost laser processing of thiol-based SAMs. Also parallel laser processing, e.g., by using micromirror displays, appears to be feasible.

## Experimental

Au-coated Si and glass supports from commercial suppliers were used as substrates (Albert PVD, Phasis). Si (100) wafers and borosilicate glass slides were chosen as the support materials. A thin Ti film, thickness ≤3 nm, served as an adhesion layer. For the experiments, the substrates were cut into pieces of about 10 × 10 mm^2^ in size. For coating with alkanethiol SAMs, all substrates were cleaned with ethanol (p.a., VWR Prolabo) and piranha solution (5 min), a 3:1 mixture of 96% sulfuric acid (suprapur, Merck) and 30% hydrogen peroxide (p.a., AppliChem), rinsed in deionized water (18 MΩ·cm Millipore), dried in a stream of high purity argon (5.0, Air Liquide) and then immersed into a 1 mM solution of 1-hexadecanethiol (HDT, ≥95%, Fluka) in degassed ethanol in a glove box at room temperature for 18 h. Subsequently, the substrates were rinsed in ethanol and dried with argon. All subsequent experiments were carried out immediately after coating.

Photothermal patterning was carried out under ambient conditions using a continuous-wave laser setup [11]. Briefly, the beam of a diode-pumped solid state (DPSS) laser operated at λ = 532 nm was focused onto the sample by means of a standard microscope objective with a numerical aperture of 0.25 (10×, Olympus). The 1/e laser spot diameter *d*_1/e_ obtained in this way was 2 µm. An acousto–optical modulator was used to chop the laser beam and adjust the laser power. The incident laser power *P* on the samples was measured on a commercial power meter with a thermal sensor (PM3Q Field Mate, Coherent).

**Figure 8:**
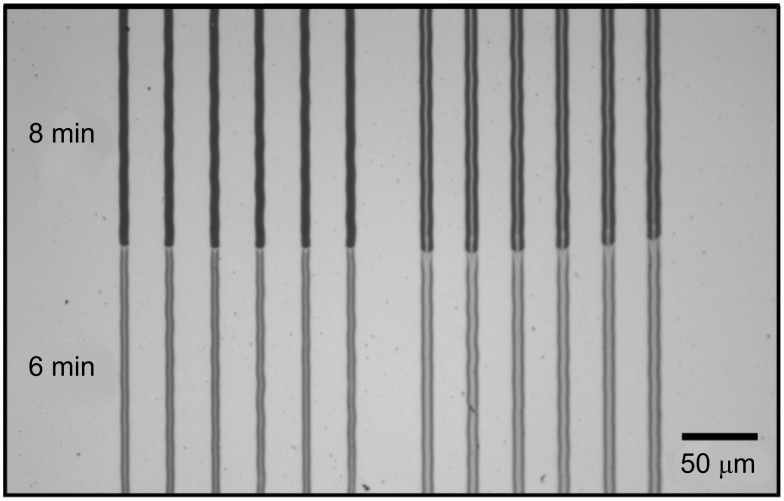
Optical micrograph of a laser-patterned HDT SAM on a Au/glass substrate exposing a 10 nm thick Au layer after wet-chemical etching. The micrograph displays surface areas that have been dipped into the etchant for 6 min (bottom) or 8 min (top).

After laser processing the patterns were transferred into the gold film by selective etching [29]. For this purpose, the patterned samples were immersed in a solution of 0.1 M K_2_S_2_O_3_ (>98%, Fluka), 1.0 M KOH (p.a., Merck), 0.01 M K_3_Fe(CN)_6_ (99%, Sigma Aldrich), and 0.001 M K_4_Fe(CN)_6_ (purum, 99%, Riedel de Haën) at room temperature. For each substrate type, the immersion time was adjusted in order to completely dissolve the Au film in the laser-depleted surface areas and to minimize widening of the structures due to the isotropic etching process. For this purpose, line patterns were fabricated on a given sample type. Subsequently the laser-patterned sample was stepwise dipped into the etching solution by employing a stepper motor stage. This allows one to test distinct immersion times on a single sample (Figure 8). After etching, the samples were rinsed in deionized water and blown dry with argon.

For the characterization of bare and HDT-coated substrates, UV–vis spectroscopy, laser reflectance and transmittance measurements, contact angle measurements and infrared reflection–absorption spectroscopy (IRRAS) were used. UV–vis spectra were measured with a Perkin Elmer UV–vis spectrometer (Lambda 950). Laser reflectance and transmittance measurements were carried out at λ = 532 nm by using the DPSS laser of the patterning setup and a power meter with a thermal sensor (cf. above). Static water contact angles were measured with an OEG SURFTENS universal goniometer. Infrared spectra were collected with a Bruker spectrometer (Vertex 70) equipped with a variable-angle reflection accessory (A513). A polarizer was placed in front of the sample in order to measure spectra with p-polarized light. The angle of the incident light was set to 85° with respect to the surface normal. The spectra were taken at a resolution of 4 cm^–1^ by using 1024 scans and were referenced to a clean gold sample without any further data manipulation.

For characterization of patterned samples, optical microscopy (BX41TS, Olympus) and AFM (Autoprobe CP from Veeco) were used. AFM images were recorded in contact mode with standard cantilevers. Width measurements were not corrected for tip-size effects and refer to values measured at half depth.
